# S1P-S1PR1 Signaling: the “Sphinx” in Osteoimmunology

**DOI:** 10.3389/fimmu.2019.01409

**Published:** 2019-06-25

**Authors:** Lan Xiao, Yinghong Zhou, Thor Friis, Kenneth Beagley, Yin Xiao

**Affiliations:** ^1^Institute of Health and Biomedical Innovation, Queensland University of Technology, Brisbane, QLD, Australia; ^2^The Australia-China Centre for Tissue Engineering and Regenerative Medicine, Queensland University of Technology, Brisbane, QLD, Australia; ^3^Key Laboratory of Oral Medicine, Guangzhou Institute of Oral Disease, Stomatology Hospital of Guangzhou Medical University, Guangzhou, China

**Keywords:** osteoimmunology, sphingosine 1-phosphate (S1P), sphingosine 1-phosphate receptor-1 (S1PR1), bone remodeling, immunomodulation

## Abstract

The fundamental interaction between the immune and skeletal systems, termed as osteoimmunology, has been demonstrated to play indispensable roles in the maintenance of balance between bone resorption and formation. The pleiotropic sphingolipid metabolite, sphingosine 1-phosphate (S1P), together with its cognate receptor, sphingosine-1-phosphate receptor-1 (S1PR1), are known as key players in osteoimmunology due to the regulation on both immune system and bone remodeling. The role of S1P-S1PR1 signaling in bone remodeling can be directly targeting both osteoclastogenesis and osteogenesis. Meanwhile, inflammatory cell function and polarization in both adaptive immune (T cell subsets) and innate immune cells (macrophages) are also regulated by this signaling axis, suggesting that S1P-S1PR1 signaling could aslo indirectly regulate bone remodeling *via* modulating the immune system. Therefore, it could be likely that S1P-S1PR1 signaling might take part in the maintenance of continuous bone turnover under physiological conditions, while lead to the pathogenesis of bone deformities during inflammation. In this review, we summarized the immunological regulation of S1P-S1PR1 signal axis during bone remodeling with an emphasis on how osteo-immune regulators are affected by inflammation, an issue with relevance to chronical bone disorders such as rheumatoid arthritis, spondyloarthritis and periodontitis.

## Introduction

Skeletal bone undergoes a life-long and continuous renovation termed “bone remodeling,” a process that is necessary for bone homeostasis and consists of osteoclasts-driven bone resorption and osteoblasts-driven bone formation (1). Osteoclasts and osteoblasts—derived from immune progenitor cells and mesenchymal stem cells (MSCs), respectively—are linked *via* immune modulators and are the fundamental cell types of these two interconnected systems. Osteoimmunology, a term first coined at the beginning of this century (2), was identified over forty years ago (3), and describes the interaction between cells from the immune and skeletal systems. The realm of osteoimmunology has revealed a complex system of mutual regulation existing between immune cells and bone cells. This relationship sees the immune response greatly affecting osteoclast-osteoblast coupling, thus mediating the balance between bone resorption and formation, whereas, at another level, cells from the skeletal system have a profound effect on the differentiation and function of immune cells.

Sphingosine is one of the most important sphingolipid metabolites (4–6). It is named after the Sphinx, a mythical creature of Greek mythology famed for its mysterious features (7). Phosphorylation of sphingosine forms the pleiotropic and bioactive lipid sphingosine-1-phosphate (S1P) (8). S1P is produced by various cell types, which acts not only as an intracellular second messenger, but also an extracellular first messenger in both an autocrine and paracrine manner. It does this by binding with a class of G-protein-coupled receptors, known as sphingosine-1-phosphate receptors (S1PRs), of which there are currently five known subtypes, S1PR1 through to S1PR5 (9). Of these receptors, S1PR1 is expressed in most mammalian cell types and considered to be multifunctional in many biological processes. S1P-S1PR1 signaling has long been addressed as a key regulator of the immune response, due to its involvement in the chemotaxis, activation, differentiation, and function of immune cells (9–13). The elevated concentration of S1P, coupled with an up-regulation of S1PR1 expression locally within inflammatory tissues in many diseases, as well as the therapeutic effects of S1PR1 modulators, is an indication of the important role of S1P-S1PR1 signaling in inflammation (8, 13).

S1P-S1PR1 signaling is primarily thought to be a catalyst of inflammation and thereby inducing osteoclastogenesis; however, the fact that this pathway is also active during bone regeneration suggests an enigmatic and rather intriguing role in bone remodeling (14, 15). In this review, we will seek to highlight the interactions between the immune and skeletal systems, how these interactions affect bone remodeling, and what is known about the role of S1P-S1PR1 signaling in the emerging field of osteoimmunology.

## The Function of S1P and its Receptor S1PR1

Sphingolipids are a key component of mammalian cell membranes and are metabolized in response to certain stimuli (4, 5). Sphingolipids are *de novo* biosynthesized from serine and palmitate in the endoplasmic reticulum (ER) (4, 5, 16, 17). The condensation of sphingolipids (*via* the action of serine palmitoyl transferase, SPT) forms 3-keto-dihydrosphingosine (16, 17), which is reduced to dihydrosphingosine, then subsequently acylated by (dihydro)-ceramide synthase (also known as Lass or CerS) to form dihydroceramide (18). The desaturation of dihydroceramide forms ceramides (19), the central player in sphingolipid metabolism (20), which could be deacylated by ceramidases (CERase) to produce sphingosines (21, 22). Sphingosine could be salvaged through reacylation, a process termed as “salvage pathway” which leading to ceramide regeneration; or it can be phosphorylated to form the multifunctional bioactive lipid S1P, which mediates a number of cellular processes, such as cell proliferation, survival, differentiation, migration, as well as cytokine and chemokine production (4, 5, 20, 23). S1P can be reversibly dephosphorylated to sphingosine by intracellular S1P phosphatases (SPPs) and extracellular lipid phosphate phosphatases, or irreversibly degraded by S1P lyase (SPL) (20, 24–27). In most mammalian cells, S1P levels are held in check by the actions of SPL and SPPs. SPL inhibition *via* both genetic and pharmacological tools results in tissue S1P accumulation *in vivo* (28). The exception is platelets, which lack SPL (29), and erythrocytes, which lack both SPL and SPPs (30). This absence explains why, under normal physiological conditions, circulating S1P levels are significantly higher (μM range) in peripheral blood than in solid tissues. S1P is also maintained at relatively high levels (>100 nM) in the lymphatic circulation, which is mainly due to the presence of lymphatic endothelial cells (31–33). Cells from the macrophage-monocyte lineage are also important producers of S1P (34).

The phosphorylation of sphingosine is performed by sphingosine kinases 1 and 2 (SPHK1 and SPHK2) (35). SPHK1 is mainly present in the cytoplasm which, after being activated by certain stimuli, is translocated to the cell membrane where it catalyzes sphingosine phosphoration (36). On the other hand, SPHK2 distributes not only in cell membrane, but also in organelles such as the ER, mitochondria, as well as in nucleus, which providing S1P for essential cellular processes, such as respiration, histone acetylation, and gene expression (37–39). For example, S1P is reported to regulate gene expression through modulating HDAC1 and HDAC2 activity (38, 40). Intracellular S1P also plays an essential part in tumor-necrosis factor-α (TNF-α) triggered NF-κB signaling *via* targeting TNF receptor-associated factor 2 (TRAF2), therefore participating the inflammatory, anti-apoptotic and immune processes (41). Once S1P is generated, it could be transported to activate its receptors, therefore functioning in a paracrine and/or autocrine manner (42, 43). This “inside-out relocation” of S1P is indispensable of special transports, as the polar head group in S1P makes it unable to move through the hydrophobic mammalian cell membranes (44). Transports such as the ATP-binding cassette (ABC) transporters family members have been demonstrated to facilitate S1P transporting in erythrocytes, platelets, and mammalian cells in an ATP-dependent manner (42, 45–49). Another transport, major facilitator superfamily transporter 2b (Mfsd2b) has also been found to play essential roles in exporting S1P in erythrocytes and platelets (50, 51). Especially, the transport spinster homolog 2 (SPNS2) is considered as a major regulator in S1P secretion in mammalian cells in a non-ATP dependent manner, which therefore playing essential roles in immune cell development and trafficking, as well as bone homeostasis (43, 52–57). Under inflammatory conditions, SPHK1 is abnormally activated to produce high levels of S1P, which is released into the local microenvironment. Inflammatory cytokines such as TNF-α, IL-1β, and interferon-γ (IFN-γ), have been shown to induce SPHK1 in an extracellular signal regulated kinase (ERK) signaling-dependent manner (38, 41, 58–60), and this partially explains the high S1P levels in the inflammatory tissues (61). Furthermore, inflammation is accompanied by vascular leakage, which may allow S1P to permeate from blood to tissues thereby raising the S1P concentrations within the inflammatory tissues (62).

**Figure 1:**
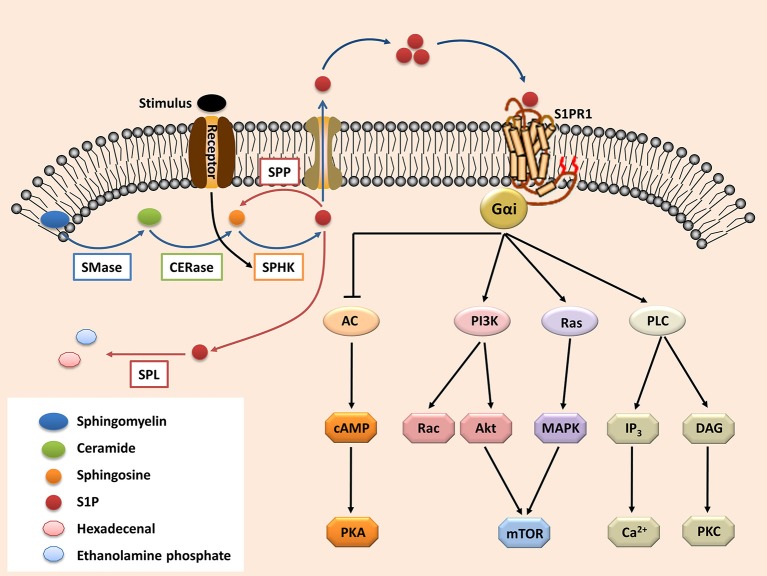
The S1P-S1PR1 signaling. Sphingolipid (derived from cell membrane) is cleaved (by sphingomyelinases, SMase) to ceramide. Ceramide is then deacylated by ceramidases (CERase) to produce sphingosine. S1P is produced by phosphorylation of sphingosine, which is mediated by SPHKs (SPHK1 and SPHK2, which can be activated by certain stimulus). S1P can be reversibly degraded by S1P phosphatases (SPPs), or irreversibly degraded by S1P lyase (SPL). On the other hand, S1P can be transported outside the cells and acts in the autocrine or paracrine manners to activate its receptor S1PR1. The S1PR1 then activates its down-stream signal cascades and therefore regulates diverse cell activities. S1P, sphingosine-1-phosphate; S1PR1, sphingosine-1-phosphate receptor 1; PLC, phospholipase C; PI3K, phosphoinositide 3-kinase; AC, adenylyl cyclase; Ras, Ras GTPase; Rac, Rac GTPase; MAPK, mitogen-activated protein kinase; cAMP, cyclic adenosine monophosphate; mTOR, mammalian target of rapamycin; PKA, protein kinase A; PKC, protein kinase C; DAG, diacylglycerol; IP_3_, Inositol trisphosphate.

The secreted S1P regulates pleiotropic biological functions by binding with its receptors (63). Upon activation, the S1P receptors couple with diverse heterotrimeric G-protein subunits (known as Gαi, Gαq/11, and Gα12/13), thereby directing different downstream signaling pathways (64). S1PR1 is the most widely expressed S1P receptor in most tissues, such as the lungs, brain, and especially immune organs (65–67). Following activation by S1P, S1PR1 interacts with Gαi, which then regulates the downstream signaling molecules (Figure 1), such as phospholipase C (PLC), phosphoinositide 3-kinase (PI3K), Ras guanosine triphosphatase (GTPase) and adenylyl cyclase (AC) (9, 68). These molecules subsequently activate their downstream signaling pathways (Figure 1), including Rac GTPase, mitogen-activated protein kinase (MAPK), Akt, and mammalian target of rapamycin (mTOR) (6, 9, 68, 69).

S1PR1 has a key role in the development of the vascular system and is highly expressed in differentiating endothelial cells (70). It is required to maintain the integrity of endothelial cell barrier and thus regulates vascular permeability responses, especially under inflammatory conditions (71). When SPHK1 is induced by inflammation, it enhances S1P production in endothelial cells, which then acts in a feed-forward manner to stimulate more S1PR1 expression, counteracting the increased permeability caused by pro-inflammatory mediators e.g., lipopolysaccharides (LPS), thereby preventing otherwise lethal cell-leakage in response to inflammation. The indispensable role of S1PR1 in vascular network stability has been further demonstrated by global *S1pr1* gene deletion, which results in defective vascular maturation and then embryonic lethality (70). Specific *S1pr1* deletion in endothelial cells results in deformities in the primary vascular plexus (angiogenic hypersprouting), limited blood flow, and vascular leakage (72–75). In epithelial cells, S1PR1 maintains cell barrier integrity and initiates the immune defense against the invading pathogens (76). S1PR1 is expressed in MSCs and regulates cell migration, proliferation, differentiation, and survival (77), whereas in osteoclast- and osteoblast-precursor cells S1PR1 expression is associated with their differentiation (78), further testament to its role in bone remodeling.

## The Regulatory Roles of S1P-S1PR1 Signaling in Bone Remodeling

### Bone Remodeling and Osteoclast-Osteoblast Coupling

Osteoclasts and osteoblasts are the major players in the bone remodeling process. The hematopoietic stem cells (HSCs)-derived osteoclasts are considered as the major type of cells responsible for bone resorption (79). Osteoclastogenesis depends on receptor activator of nuclear factor-kappa B ligand (RANKL), a cytokine in the TNF family (80). RANKL activates its cognate receptor, receptor activator of nuclear factor-kappa B (RANK), initiating osteoclastogenic signals (Figure S1). The RANKL-RANK axis, together with the downstream NF-κB signaling pathway, is indispensable in osteoclastogenesis (81, 82). Another key factor in osteoclast formation is macrophage colony-stimulating factor (M-CSF), which is critical in regulating survival and proliferation of osteoclast precursors (83).

Osteoblasts are the major producer of RANKL and M-CSF (84), indicating that osteoclasts and osteoblasts are related “coupling” cells that link osteoclastogenesis to osteogenesis. Osteoblasts are derived from MSCs and are the main cell type responsible for bone formation (79). Factors such as alkaline phosphatase (ALP), runt-related transcription factor 2 (RUNX2), osteocalcin (OCN), and the Wnt/β-catenin and TGF-β signaling pathways, as well as the signal transducer and activator of transcription 3 (STAT3) signaling, are considered to be crucial in osteogenesis (85–89). Besides RANKL and M-CSF, osteoblasts also produce osteoprotegerin (OPG), which, conversely, acts as a decoy receptor of RANKL and thereby impeding osteoclastogenesis (90). Hence, osteoclasts and osteoblasts are interconnected by the RANKL/RANK/OPG axis, with the ratio of RANKL to OPG determining the balance between bone resorption and formation.

Bone remodeling is a strictly regulated process that must maintain bone formation at a rate equal to that of bone resorption (2). Skeletal pathologies arise when this balance is disrupted. The most common one of such disorders is when bone remodeling is skewed toward resorption—that is, when osteoclastogenesis is aberrantly stimulated so the rate of bone resorption exceeds bone formation, resulting in a net bone loss, as seen in inflammatory diseases, such as rheumatoid arthritis (RA) (91), periodontitis (92), and apical periodontitis (93).

### The Roles of S1P-S1PR1 in Bone Remodeling

S1P has been found to induce both osteoclastogenesis and osteogenesis, a dual role that makes S1P-S1PR1 signaling more intriguing.

#### S1P-S1PR1 Signaling in Osteoclastogenesis

Together with its ligand S1P, S1PR1 directs chemotactic migration of osteoclast precursors *in vitro* and *in vivo*. S1P-S1PR1 signaling is thought to regulate osteoclast precursor trafficking to and from bone surface, where the precursor cells fuse and differentiate into osteoclasts, a process which dynamically regulates bone mineral homeostasis and osteoclastogenesis (78). S1PR1-dependent chemo-attraction is only activated when S1P concentration is comparably low. High concentrations of S1P activates the S1PR2 on the precursor cells and triggers an S1PR2-dependent chemo repulsion (94). This mechanism partially explains how these precursor cells are retained in bone marrow, where lower levels of S1P are found than in the peripheral blood. S1PR1 and S1PR2 act in a concerted manner to regulate osteoclast precursors egressing from bone marrow into circulation, depending on the relative concentrations of S1P. It is also found that the active form of vitamin D, 1,25-D, and its clinically used analog, eldecalcitol (ELD), effectively reduce bone resorption *via* inhibiting S1PR2 in circulating osteoclast precursors, as S1PR2-blockage directs the migration of osteoclast precursors from bone surface to blood. This study reveals the pharmacologic effect of vitamin D analog in therapy against osteoporosis (95), suggesting that the “S1PR1-S1PR2 concert” should be considered as a therapeutic target for diseases with bone loss. During RANKL-mediated osteoclast differentiation, the activity of SPHK1 is significantly enhanced and increases production of S1P by the precursor cells. Conversely, inhibition of SPHK1 leads to suppression of osteoclastogenesis (34).

#### S1P-S1PR1 Signaling in Osteogenesis

Although S1P is found to induce osteoclastogenesis, it also plays a positive role in osteogenesis. In the process of BMP-2-mediated osteoblast differentiation, S1P significantly induces ALP activity and the expressions of key bone formation markers, such as OCN and RUNX2. Enhanced BMP-2/SMAD signaling is the result of MEK (mitogen-activated protein kinase kinase) 1/2-ERK1/2 pathway activation (14). Another study indicates that S1P-S1PR1 signaling activation in osteoblasts mediates the activation of PI3K/Akt signaling and therefore inhibits glycogen synthase kinase-3β (GSK-3β), which leads to induced nuclear translocation of β-catenin, a key process in osteogenesis (96). S1P has also been found to induce RUNX2 expression in osteoblasts and thereby improve osteogenesis *in vitro* and *in vivo*, which is achieved through S1PR2-dependent activation of Smad1/5/8 signaling (97). Conditioned medium from osteoclasts can induce osteogenesis and is thought to be due to Wnt10b, BMP-6, and S1P secreted into the medium. And whereas S1P and BMP-6 can trigger the migration of pre-osteoblasts toward bone resorption sites, S1P can also induce osteogenic differentiation of the same cells by activating S1PR1, a finding that becomes apparent when S1PR1 is blocked (15). These properties of S1P-S1PR1 signaling to some degree explain how bone formation is initiated following bone resorption. Accordingly, hormone calcitonin (CT) has been found to block S1P secretion of osteoclast *via* SPNS2 inhibition, which consequently results in decreased bone formation *in vivo* in a S1PR3-dependent manner (55). In a more recent study, induced expression and activity of SPHK1 and SPHK2 have been observed during the *in vitro* osteoblast differentiation, accompanied with enhanced *Spns2* gene level, as well as increased S1P secretion. Blockage of SPHK1 or SPHK2 results in retarded osteogenic differentiation and mineralization, suggesting the indispensable role of S1P signaling in osteogenesis (54).

#### S1P-S1PR1 Signaling in Osteoclast-Osteoblast Coupling

Interestingly, intracellular S1P, which is produced during osteoclastogenesis, also inhibits this process, by suppressing p38-MAPK signaling, a key signaling pathway downstream of RANK (Figure S1). This is in contrast with extracellular S1P which has no effect on osteoclast differentiation, suggesting S1P can target cells other than osteoclasts, e.g., the coupling osteoblasts (34). S1P activates p38-MAPK and ERK signaling in osteoblasts, resulting in increased levels of cyclooxygenase-2 (COX2). COX2 induces the expression of prostaglandin E2 (PGE2), which prompts the production of RANKL by osteoblasts. RANKL binds to its receptor RANK on osteoclast precursors which promotes osteoclast differentiation and S1P secretion, thereby setting up a feed-forward loop for osteoclastogenesis.

Cathepsin K (CSTK) is an enzyme that is involved in bone degradation which, when specifically deleted in osteoclast lineage by targeted *in vivo* gene modification, results in a condition characterized by an increased number of osteoblasts and bone formation, as well as an increased number of dysfunctional osteoclasts and impaired bone resorption (98). The *in vitro* analysis of primary osteoblasts showed enhanced ALP activity and osteogenic potential, as well as increased RANKL/OPG ratio. Osteoclasts from *CSTK*-knockout mice presented with up-regulated expression of SPHK1 and increased S1P production leading to a higher RANKL/OPG ratio of the primary osteoblasts, which in turn increased the number of osteoclasts. The antagonist of S1PR1 and S1PR3 reduced the osteogenic ability of osteoblasts induced by the conditioned medium of *CSTK*-deficient osteoclasts, suggesting the enhanced *in vivo* osteogenesis was due to the activation of S1PR1 and S1PR3 (98).

In a more recent study, S1P degradation was blocked *via* SPL inhibition (through both genetic and pharmacological means) *in vivo*, and this resulted in increased bone mass and enhanced bone strength, accompanied with induced OPG expression and reduced osteoclastogenesis in mice (28). Further research revealed the role of S1P-S1PR2 under this phenomenon. In osteoblast, S1P-S1PR2 signaling played a significant role in bone remodeling, which not only promoting the osteogenic differentiation, but also inducing OPG production *via* p38–GSK3β-β-catenin and Wnt5A–LRP5 pathways, suggesting S1P-S1PR2 signaling should improve bone formation while limiting bone resorption. Accordingly, SPL inhibition ameliorated osteoporosis in OPG-deficient mice through inducing the activity and mineralization of osteoblast while reducing osteoclastogenesis. In addition, S1PR2-deficience resulted in osteopenia in mice, accompanied with reduced OPG expression and retarded differentiation of osteoblast (28). These results indicate that similar to S1PR1, S1P-S1PR2 signaling also acts as a coupling factor between osteoclast and osteoblast. However, S1PR2 activation leads to increased OPG production, which possibly neutralizing S1PR1-mediated RANKL expression and hence osteoclastogenesis. It is presumed that S1PR1-S1PR2 may act in a balanced way to maintain physiological bone remodeling, while this balance might be destroyed under pathological conditions such as inflammation, which needs further investigation. From these studies, it could be concluded that S1P acts as a coordinator between bone resorption and formation, which, in combination with its positive effects in both osteoclastogenesis and osteogenesis, suggesting a complicated role of this signaling in bone remodeling.

## The Immunomodulatory Role Of S1P-S1PR1 Signaling in Osteoimmunology

The balance of bone remodeling is maintained by the immune system, which, therefore, links the skeletal, and immune systems together. As a key regulator of the immune system, the S1P-S1PR1 signaling could be postulated to indirectly impact bone remodeling by the immunomodulation, indicating its enigmatic role in osteoimmunology.

### Osteoimmunology

**Figure 2:**
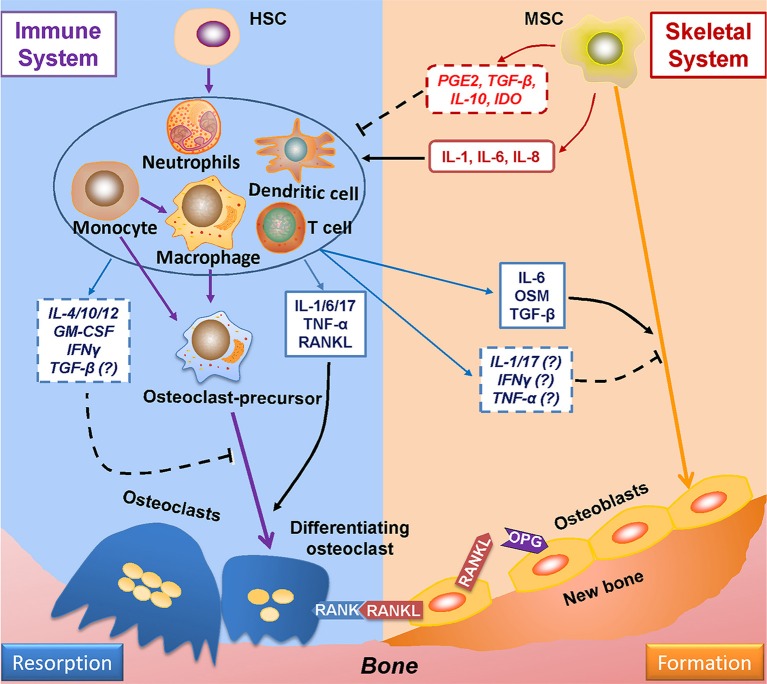
Mutual regulations between the immune and skeletal systems. The two major players in bone remodeling—osteoclasts and osteoblasts are coupled through the RANKL-RANK-OPG axis: osteoblasts-derived RANKL combines with its ligand RANK in osteoclasts and plays an indispensable role in osteoclastogenesis; OPG (which is also derived from osteoblasts) reduces osteoclastogenesis by impairing the RANKL-RANK signaling. The immune system greatly takes part in osteoclastogenesis by producing RANKL; also, the immune-related factors either affect pre-osteoclasts, or interacts with osteoblasts to induce RANKL production to regulate osteoclastogenesis. The immune-derived regulators also affect the process of osteogenesis. On the other hand, the progenitor cells of the skeleton system—MSCs suppress immune response either by cell to cell intact or by secreting functional regulators; whereas under certain conditions, MSCs upon TLR4 stimulation secret factors which induce immune response. RANKL, receptor activator of nuclear factor factor-kappa B ligand; RANK, receptor activator of nuclear factor-kappa B; OPG, osteoprotegerin.

Evidence of the relationship between the immune and skeletal systems became apparent with the finding that IL-1, secreted by antigen-stimulated immune cells, plays a positive role in osteoclastogenesis (99). Since then, many more studies have demonstrated the role of immune system on bone remodeling (Figure 2) (100). Furthermore, cells derived from skeletal system, such as MSCs, are capable of regulating immune responses (101). Such findings gave birth to osteoimmunology, a field that is concerned with interactions between immune and skeletal systems, within which the cells from each system are correlated through a variety of factors and signaling pathways such as S1P-S1PR1.

#### Regulation of Bone Remodeling by Immune System

The adaptive immune cells—T-helper cells—play a critical role in bone remodeling by producing RANKL, the key factor in osteoclastogenesis, and also other factors that regulate bone metabolism. Cytokines derived from type 1 helper T (Th1) cells, such as IFNγ and granulocyte-macrophage colony-stimulating factor (GM-CSF), suppress osteoclastogenesis by interrupting the RANK signaling (Figure 2) (102–105). However, it is also reported that GM-CSF facilitates the fusion of pre-osteoclasts into multinucleated osteoclasts, suggesting a fundamental role of GM-CSF in the function of osteoclasts (106, 107). In addition, GM-CSF derived from breast tumor cells has been found as responsible for osteolytic bone metastasis *in vivo* (107). Other cytokines derived from type 2 helper T (Th2) cells, such as interleukin-4 (IL-4) and IL-10, also inhibit RANK signaling and osteoclast differentiation (108–110). IL-6, which is produced by Th2 cells and M1 macrophages, triggers osteoclastogenesis by promoting RANKL production, as well as stimulating IL-1 production, which amplifies the inflammatory response (111–113). IL-6 also induces the differentiation of type 17 helper T (Th17) cells, which secrete the pro-inflammatory cytokine IL-17 (114, 115), and in turn promote RANKL secretion and osteoclastogenesis (116, 117). The immune-suppressive regulatory T (Treg) cells (118), inhibit osteoclastogenesis in a direct cell-to-cell contact-dependent manner, by binding of cytotoxic T-lymphocyte-associated protein 4 (CTLA-4) on Treg cells with CD80 and CD86 on osteoclast precursors; Treg cells also reduce osteoclastogenesis by secreting IL-4 and IL-10 (119). Another Treg cell-derived factor, TGF-β, has pleiotropic effects on osteoclastogenesis. On one hand, TGF-β can induce osteoclast differentiation by promoting RANK expression and regulate activator protein 1 (AP-1) signaling (120, 121), a key downstream effector of RANK (Figure S1). However, in osteoclast-osteoblast co-cultures, TGF-β can also suppress RANKL expression in osteoblasts, effectively applying the brakes on osteoclastogenesis (120).

Cells from the innate immune system also contribute to the regulation of osteoclastogenesis. Macrophages, the major components of innate immunity, constitute three sub-populations of cells: (1) non-activated M0 macrophages; (2) pro-inflammatory M1 macrophages, which are classically activated by LPS or Th1 cell cytokines such as IFNγ; and (3) M2 macrophages, which is alternatively activated by Th2 cell cytokines, such as IL-4 or IL-13, and are classified as anti-inflammatory macrophages (122–125). Macrophages are precursors of osteoclasts (126) and secrete factors that actively affect osteoclastogenesis. M1 macrophages express IL-1α and IL-1β which activates RANK signaling thereby inducing osteoclastogenesis, under both physiological and pathological conditions (127, 128). M1 macrophages also express TNF-α, which stimulates osteoclast differentiation by activating the NF-κB signaling (129, 130). Moreover, TNF-α promotes RANKL expression of osteoblasts to induce osteoclastogenesis (131, 132). On the contrary, M2 macrophages-derived IL-10 (133) is a negative regulator of osteoclastogenesis (110).

These immune-derived factors also participate in the regulation of osteogenic process. Originated from Treg cells and M2 macrophages, TGF-β has been identified as a crucial factor in osteoblast differentiation and mineralization (134). M2 macrophages also recruit MSCs (osteoblast precursors) by producing the transmembrane glycoprotein Osteoactivin (OA)/Glycoprotein non-metastatic melanoma protein B (GPNMB) (135). Interestingly, some pro-inflammatory factors, known as osteoclastogenic promoters, have also been found to induce osteogenesis. For instance, IL-6 can enhance ALP activity *in vivo via* STAT3 signaling, a further indication of the ability of IL-6 to affect osteogenesis (136–140). Originated from M1 macrophages, oncostatin M (OSM) facilitates osteogenesis by activating RUNX2 *via* STAT3 signaling pathway. Studies with OSM or OSM receptor (OSMR) deficient mice show reduced bone healing, evidence for its critical role in osteogenesis (141, 142). There are studies indicate that IL-1 (143, 144), IL-17 (145, 146), and TNF-α (147) play positive roles in bone formation *in vitro* and *in vivo*, however, conflicting results exist (Table 1).

**Table 1:** Effects of immune cells on bone remodeling.

**Immune cells**	**Main functional factors**	**Effects on osteoclastogenesis**	**Effects on osteogenesis**
M1 macrophages	IL-1	Activation (127, 128)	Activation (143, 144)/Inhibition (148, 149)
	TNF-α	Activation (129–132)	Activation (147, 150)/Inhibition (148, 149)
	OSM	Activation (151, 152)	Activation (141, 142)
M2 macrophages	IL-10	Inhibition (110)	Activation (153)/Inhibition (154, 155)
	TGF-β	Dural (120, 121)	Activation (134)
Th1 cells	IFNγ	Inhibition (105)	Activation (156, 157)/Inhibition (238)
	GM-CSF	Activation(106, 107)/Inhibition (102–104)	Activation (158)
Th2 cells	IL-4	Inhibition (108, 109)	Inhibition (159)
	IL-6	Activation (111–113)	Activation (136–140)
Th17 cells	IL-17	Activation (114, 115)	Activation (145, 146)/Inhibition (160)
Treg cells	CTLA-4	Inhibition (119)	–

*CTLA-4, cytotoxic T-lymphocyte-associated protein 4; GM-CSF, granulocyte-macrophage colony-stimulating factor; IFNγ, interferon-γ; IL, interleukin; OSM, oncostatin M; TGF-β, transforming growth factor-β; Th, T helper; TNF-α: tumor necrosis factor α; Treg, regulatory T*.

Accumulating evidences indicate that macrophages play an indispensable role in bone formation. The bone residential macrophages are required in osteogenesis and are, more importantly, also needed for the maintenance of bone-forming surfaces. Both M1 and M2-derived secreted factors are found to promote osteogenesis, especially M1-derived OSM (142). Interestingly, RANKL is found to induce a M1-like macrophage phenotype; this M1-like macrophage infiltration appears during the early stage of bone repair and is identified to facilitate osteogenesis (167). Furthermore, the conversion of M1 to M2 macrophages significantly improves mineralization of the co-cultured osteoblasts *in vitro* (168). This is consistent with the *in vivo* macrophage polarization during bone healing, that the infiltration of M1-like macrophages during the early inflammatory phase is indispensable for bone healing, while the M2-like macrophage infiltration becomes dominant in the later stage of bone repair (167). It can be presumed that the transient activation of M1 macrophages are essential for the early osteoblast activation, while M2 macrophages are indispensable for the later mineralization. Especially, cells from the macrophage—monocyte lineage are considered as important source of S1P (28), a crucial regulator in bone remodeling as discussed above, suggesting that macrophage-derived modulation on bone remodeling might also due to S1P-S1PR1 signaling, which needs further study in the future.

#### Immune-Regulation Mediated by Cells From Skeletal System

The skeletal system exerts a regulatory effect on the immune system *via* the actions of MSCs, which are capable of suppressing the differentiation and function of effector immune cells, such as Th1, Th17, and M1. MSCs can inhibit differentiation of M0 macrophages to dendritic cells (DCs) and suppress their maturation and function. MSCs also induce macrophage polarization to the M2 phenotype and interfere with T cell proliferation, cytokine production and polarization, in particular the promotion of Treg cell differentiation (101, 169–172). The immune-suppressing functions of MSCs are achieved either through direct cell-cell contact or secretion of soluble immune-modulators, some of which are produced constitutively while others are produced in response to inflammatory factors or activated immune cells (173). Direct cell-cell contact suppression is achieved through the programmed death 1 (PD-1) pathway (174), whereas immune suppressive factors include prostaglandin E2 (PGE2), TGF-β, IL-10, leukemia inhibitory factor (LIF), IL-1 receptor antagonist (IL-1RA) (173, 175). Of these factors, PGE2 is considered to be one of the most potent in MSCs' immunosuppressive arsenal, especially in term of macrophage polarization (101, 176). MSCs secrete PGE2 in response to pro-inflammatory factors, such as IFNγ or LPS (171, 177) and convert M1 macrophages to M2 phenotype (178). This process, which depends on PGE2, induces the production of immune suppressive cytokines (such as IL-10), while impeding the secretion of pro-inflammatory cytokines (such as TNF-α and IL-6), resulting in a microenvironment more suitable for tissue regeneration (171, 179). These effects of PGE2 directly affect the immune response and acts as a coupling factor between macrophages and MSCs/pre-osteoblasts in a way that facilitates osteogenesis (180).

However, when toll-like receptors (TLRs) are activated by LPS, IFN-α/γ, or TNF-α, MSCs can respond by producing pro-inflammatory cytokines (173) such as IL-1β and IL-6 and the chemokine IL-8, which attract the migration of neutrophils and augment the inflammatory response (181). It has emerged that similar to macrophages, human MSCs also polarizes into two distinct phenotypes: pro-inflammatory MSC1 and immunosuppressive MSC2 (182). TLR signaling plays an active role in this polarization, in which acute and low-level activation of TLR4 directs MSCs toward the MSC1 phenotype, whereas the TLR3 activation induces an MSC2 phenotype. The MSC1 phenotype can also be induced by IFNs or direct contact with certain pro-inflammatory cells. Polarized MSCs are thought to play roles similar to that of M1 and M2 macrophages in tissue repair (183), with MSC1s contributing to early stage inflammation and MSC2s contributing to late tissue regeneration. Of note, a recent study has found that macrophage-derived inflammatory factors could induce the RANKL production of bone marrow stromal cells through the SPHK1-S1PR1 axis (184), suggesting that S1P-S1PR1 signaling might participate in MSC polarization and therefore in turn regulate immune response.

### Roles of S1P-S1PR1 in Osteoimmunology

**Figure 3:**
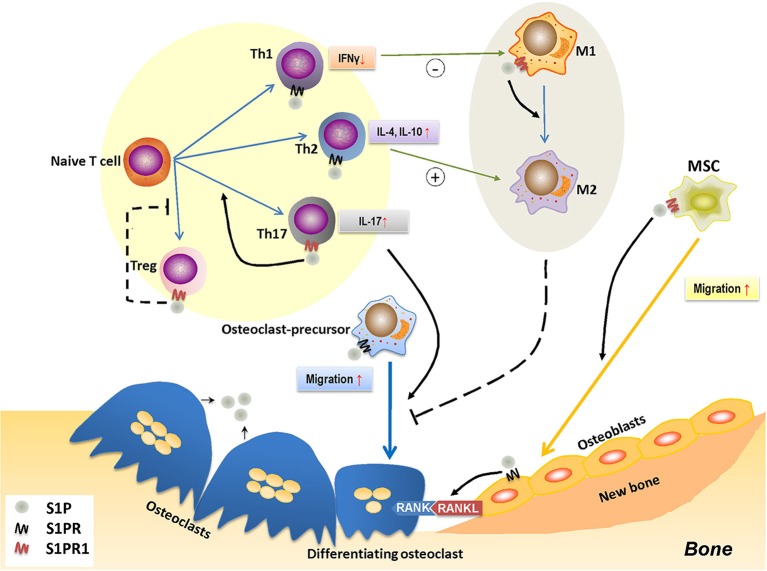
The role of S1P-S1PR1 signaling in osteoimmunology. S1P-S1PR1 signaling is greatly involved in the interaction between immune system and bone remodeling. On one hand, S1PR1 directly affects osteoclastogenesis by inducing the migration of osteoclast-precursors. The direct effect of S1P on osteoclast-precursors results in reduced osteoclastogenesis; however, it induces RANKL production of osteoblasts and facilitating the RANKL-RANK mediated osteoclastogenesis. S1P also induces the migration of MSCs and osteogenesis by activating S1PR1. On the other hand, S1P-S1PR1 signaling participates in immune regulation, which affects the polarization and function of T-helper cells. S1P-S1PR1 signaling induces the differentiation and function of Th17 cells (known as inducing osteoclastogenesis) while impedes that of Treg cells (known as reducing osteoclastogenesis); therefore facilitating osteoclastogenesis. S1P also induces the function of Th2 cells while reduces that of Th1 cells, which affects the macrophage phenotype; also, S1P directly induces the transition of M1 to M2 phenotype by activating S1PR1. This conversion of pro-inflammatory M1 macrophages to tissue-engineering M2 macrophages therefore impedes osteoclastogenesis, which might also affect osteogenesis. S1P, sphingosine-1-phosphate; S1PR, sphingosine-1-phosphate receptor(s); S1PR1, sphingosine-1-phosphate receptor 1; MSC, mesenchymal stem cell; Th1/2/17, type 1/2/17 helper T cell; M1/M2, M1/M2 macrophage; Treg, regulatory T cell; IFNγ, interferon-γ; IL-4/10/17, interleukin-4/10/17.

When S1P binds with S1PR1, it forms a complex that governs a diverse range of immune cell activities, such as cell migration, proliferation, and differentiation (185). This immunomodulatory effect is thought to be pivotal for bone remodeling (Figure 3).

S1P-S1PR1 signaling plays a decisive role in regulating the traffic and egression of immune cells, such as HSCs, DCs, macrophages (monocytes), neutrophils, mast cells, T and B lymphocytes, natural killer T (NKT) cells (78, 186–193). Under both homeostatic and pathological conditions, S1P-S1PR1 signaling is required for mature thymocytes to egress from the thymus, as are T/B cells from secondary lymphoid tissues into blood or lymph (188, 194–196). S1PR1 deficiency results in blocked lymphocyte egression, a condition known as lymphopenia (196), suggesting a vital role for S1PR1 in the timely and appropriate distribution of immune cells, a process that aids homeostasis of the immune system. During inflammation, there is a spike of the local concentration of S1P, results in activated S1PR1 and the recruitment of immune cells—such as effector T cells—to the inflamed tissues and their *in situ* retention (61), which therefore promotes the inflammatory response—a process that induces bone resorption (100).

S1P-S1PR1 signaling is also an essential modulator of immune cell differentiation and function. S1P is required for the maturation and function of DCs, which further affects the activation and polarization of CD4^+^T cells (197, 198). S1P regulates the function and especially the polarization of CD4^+^T cell subsets. S1PR1 activation in CD4^+^T cells impairs the production of IFNγ by Th1 cells, while enhance the production of Th2 cells-derived effector cytokine IL-4, thereby downregulating the Th1 cell response while upregulating that of Th2 cells (162, 163, 199, 200). On the other hand, S1P can induce the differentiation and activation of Th17 cells, as well as the production of IL-17 *in vitro* (Table 2)—both of which promote osteoclastogenesis (201). This is accompanied by reduced production of Th1 and Th2 cell-derived cytokines, a process that is considered to be S1PR1-dependent (164, 165). Furthermore, signaling through S1PR1 impedes the differentiation and function of Treg cells, the vital suppressor in immune response and osteoclast differentiation (118), by activating the downstream Akt-mTOR signaling pathway (166, 202), thereby exacerbating bone resorption (Table 2). More importantly, by enhancing RANKL production in CD4^+^T cells S1P contributes to osteoclastogenesis (203).

**Table 2:** Possible effects of S1P-derived immune-regulation on bone remodeling.

**Cell type**	**Immune-regulation of S1P**	**Possible effects on bone remodeling**	
		**Osteoclasto-genesis**	**Osteogenesis**
M1 macrophages	Differentiation↓ (161)	↓	↑
M2 macrophages	Differentiation↑ (161)	↓	↑
Th1 cells	Response↓ (162)	↓	↑
Th2 cells	Response↑ (163)	↓	↑
Th17 cells	Differentiation↑ (164, 165) Response↑ (164, 165)	↑	↑(?)
Treg cells	Differentiation↓ (165, 166)	↑	-

*Th, T helper; Treg, regulatory T*.

However, in macrophage polarization, S1P-S1PR1 signaling tends to favor differentiation to an anti-inflammatory phenotype, inducing a conversion of the M1 to M2 subset (161). The S1P-derived induction of Th2 response and IL-4 secretion may indirectly affect this process. The shift from M1 toward the M2 subset (161) could be considered as reducing osteoclastogenesis, since the M1 macrophage-derived cytokines are recognized as inducers for osteoclast differentiation (Table 1). A similar shift may also take place in osteogenesis in which M1 macrophages, indispensable during the early stages of bone repair, shift toward the M2 phenotype that is required in the later stages of bone formation (142, 168). Therefore, in contrast to its immune-inductive role in CD4^+^ T cell polarization, S1P-S1PR1 signaling has an immune-suppressive role in determining macrophage polarization, which complicates its role in bone remodeling (Table 2).

**Figure 4:**
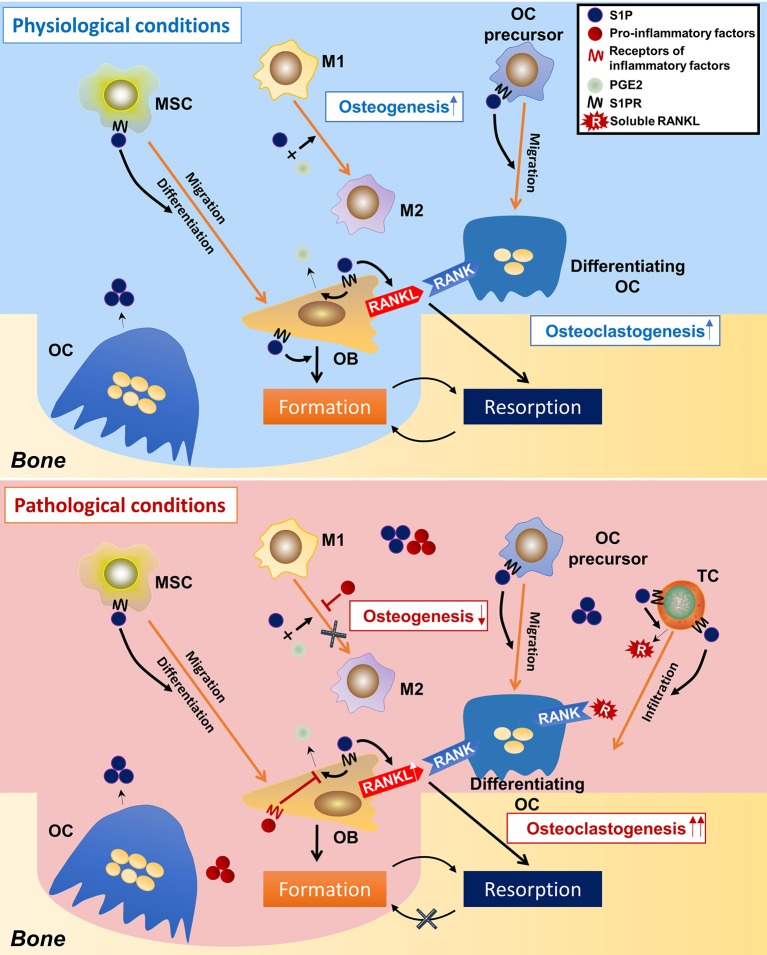
Speculations on the regulatory roles of S1P on osteoimmunology under physiological and pathological conditions. In the physiological condition, osteoclast-derived S1P initiates bone formation by triggering MSCs migration to the resorption cite and by inducing osteoblasts differentiation. During the osteogenic process, S1P also induces the production of PGE2, which, together with S1P, leads the polarization of macrophages toward the M2 phenotype, thereby facilitating bone formation. S1P also induces RANKL production of osteoblasts, as well as the migration of osteoclast-precursors, initiating a new round of osteoclastogenesis. This makes the constant remodeling and bone metastasis. However, in the pathological condition (inflammation), the over-accumulated S1P results in infiltration of inflammatory cells (i.e., T-helper cells), which not only secret large amounts of pro-inflammatory cytokines, but also produce a lot of RANKL (in stimulation of S1P), which greatly induces osteoclastogenesis. On the other hand, the pro-inflammatory factors neutralize the immune-suppressive function of S1P and PGE2 on macrophages, result in failed conversion from M1 to M2 phenotype–an unsuitable circumstance for osteogenesis. This eventually makes to the imbalance between bone resorption and formation and thereby bone loss. S1P, sphingosine-1-phosphate; S1PR, sphingosine-1-phosphate receptor(s); MSC, mesenchymal stem cell; M1/M2, M1/M2 macrophage; OC, osteoclast; OB, osteoblast; TC, T cell; PGE2, prostaglandin E2.

From these studies, a picture emerges of how S1P modulate osteoimmunology (Figure 4). Under physiological conditions, S1P secreted from osteoclasts during normal bone resorption may initiate bone formation. S1P prompts the migration and subsequent differentiation of MSCs to the resorption pits and also promotes the secretion of PGE2. The combined effect of S1P and PGE2 determines macrophage phenotype and creates a microenvironment suitable for bone regeneration. On the other hand, S1P and PGE2 induce the RANKL expression of osteoblasts. Osteoclast-precursors, which are also recruited by S1P, migrate to the resorption site where they are exposed to osteoblast-derived RANKL, which promotes their differentiation into mature osteoclasts, thus underpinning the continuous process of bone remodeling. Under pathological conditions, such as inflammation, the effects of S1P and PGE2 on macrophages are counteracted by inflammatory cytokines, which interfere with the conversion of M1 to M2 macrophages, resulting in a microenvironment that is unfavorable to osteogenesis. This is further exacerbated once the MSCs stop being immunosuppressive and exhibit a pro-inflammatory phenotype. T cells are also activated by S1P, which infiltrate in the site of resorption and secrete more RANKL into the local microenvironment. The high concentration of RANKL and inflammatory cytokines leads to a catabolic imbalance that favors bone resorption. Of note, it is still unclear whether S1P signaling leads to “normal” or “abnormal” bone formation, as elevated S1P has also been found in diseases with unwanted excessive bone formation such as spondyloarthritis (54, 204).

Taken together, the weight of evidence all points to S1P-S1PR1 signaling having a pivotal role in osteoimmunology. At one level there is a direct link between S1P-S1PR1 and osteoclast-osteoblast coupling; however, there is also an indirect link that affects bone remodeling *via* S1P-S1PR1 regulation of immune response. Under certain pathological conditions, this finely tuned system is thrown into disequilibrium resulting in an overactive immune environment where bone resorption outstrips formation.

## S1P-S1PR1 Signaling in Bone Diseases

Abnormally activated S1P-S1PR1 signaling has been observed in many diseases, such as RA, multiple sclerosis and cancer (205–207). The importance of S1P-S1PR1 signaling in osteoimmunology highlights the need to assess its roles in the pathogenesis of bone diseases. In addition, S1P regulation *via* SPL inhibition has been demonstrated to enhance bone mass and strength in a S1PR2-dependant manner *in vivo*, which also effectively ameliorating osteoporosis in S1PR2-deficient mice, suggesting S1P is a potential therapeutic target for bone diseases (28).

RA is an autoimmune disorder of the joints characterized by excessive osteoclastogenesis—the result of inflammatory immune response (206). Activated S1P-S1PR1 signaling is found in the synovial tissues of RA joints (206), which is considered to promote RANKL production of CD4^+^T cells and synoviocytes in a COX-2-dependant manner (203). The joint and bone destruction is significantly alleviated in *Sphk1*-deficient mice: the reduced circulating S1P leads to limited COX-2 expression and Th17 differentiation, with a resulting inhibition of osteoclastogenesis in inflammatory joints (208). Fingolimod, also known as FTY720, is a sphingosine analog that acts as a modulator of S1P-S1PR1 signaling, which has been clinically used in treatment against multiple sclerosis (209). FTY720 is phosphorylated by SPHK2 (FTY720-P) *in vivo* to gain high affinity to S1PR1 (210, 211). Although both S1P and FTY720-P induce S1PR1 internalization (212, 213), the endocytosed S1PR1 following S1P binding is eventually recycled back to cell surface (212); while the endocytosed S1PR1 induced by FTY720-P is then irreversibly degraded (184, 213–217), resulting a pharmacologic deletion of S1PR1 from cell surface (218). FTY720 has been demonstrated to be effective in a mouse RA model, which inhibited the infiltration of effector CD4^+^T cells and reduced IL-6 and TNF-α expression in synovial fibroblast cells (219). Similar results have been found in adjuvant-induced arthritis (AA) and collagen-induced arthritis (CIA) rodent models, which were achieved *via* modulating the migration of T cells and DCs, as well as regulating T cell polarization (220–222), suggesting that S1PR1-deletion could be a pharmacological strategy for RA. Interestingly, strategies to increase S1P also showed therapeutic effects in RA animal models. SPL inhibitors, (E)-1-(4-((1R,2S,3R)-1,2,3,4-Tetrahydroxybutyl)-1H-imidazol-2-yl)ethanone Oxime (LX2931) and (1R,2S,3R)-1-(2-(Isoxazol-3-yl)-1H-imidazol-4-yl)butane-1,2,3,4-tetraol (LX2932) have been found to reduce symptoms and pathological changes in the RA mice model, which could dose-dependently decrease the numbers of circulating lymphocytes by sequestrating them in the thymus (223). In phase I clinical trial, LX2931 administration effectively decreased peripheral lymphocyte counts, suggesting it could potentially reduce local inflammation in RA patient (223). The similar effects between S1P induction and S1PR1 reduction indicate that other S1PRs such as S1PR2, which has demonstrated effects against S1PR1 (28, 94), should also be considered as therapeutic target for RA in the future.

Besides RA, S1P signaling might also participate in the pathogenesis of other arthritis such as spondyloarthritis. Spondyloarthritis (SpA) is a group of several inner-related disorders: psoriatic arthritis, arthritis related to inflammatory bowel disease, reactive arthritis, a subgroup of juvenile idiopathic arthritis, as well as ankylosing spondylitis (the prototypic subtype) (224). Spondyloarthritis is characterized by enthesopathy—inflammation at the cites (named as enthesis) where ligaments and tendons attach to the bone through fibrocartilage connections (54, 224). SpA at later stage usually results in abnormities at enthesis such as excessive bone formation, increased mineralization and fusion of bone, as well as ankyloses (54). A recent study has found that the S1P levels in serum from SpA patients are significantly induced, as compared with those from healthy donors (54, 204). S1P has also been found to induce the mineralization of primary chondrocytes and osteoblasts originated from enthesis (54). This suggests the accumulation of S1P may result in the excessive ossification in SpA, which still needs further verification (54).

S1P is also strongly associated with the pathogenesis of infection-related inflammatory bone loss, as seen in periodontitis and periapical lesions: an inflammatory condition caused by teeth-related bacterial infections that erodes alveolar bone. In a mouse periodontitis model, the ablation of SPHK1 can significantly attenuate alveolar bone loss and is accompanied by a reduction in the numbers of leukocytes and osteoclasts in the periodontal tissues (225). S1P-S1PR1 signaling is also linked to periapical lesions: an upregulation of S1PR1 positively correlates with RANKL and osteoclast expression and negatively with the number of Treg cells during the pathogenesis of periapical bone destruction (226). Further research into this phenomenon indicates that infection-induced M1 macrophages interact with osteoblast—precursors to enhance the production of S1P, which acts in an autocrine manner to activate S1PR1 on osteoblast-precursors. The activation of S1P-S1PR1 signaling results in induced RANKL production, which is partially achieved through the mTOR signaling-dependent inhibition of autophagy in osteoblast-precursors (184). These studies suggest modulation of S1P-S1PR1 signaling could be a novel therapeutic strategy for infection-induced inflammatory bone diseases.

## Future Directions and Conclusion

Although S1P has been studied for years, many questions still remain un-resolved regarding its role in bone remodeling. For instance, the actual outcome of S1P-S1PR1 signaling-derived modulation on bone remodeling is unknown, since it is found to induce both osteoclastogenesis and osteogenesis. The role of S1P-S1PR1 signaling in osteoimmunology is even more complicated, as its downstream signaling pathway, mTOR, has a dual role in immune system, that in Th cells it directs the polarization toward inflammatory phenotype, while in macrophages it directs the anti-inflammatory M2 polarization (125, 227–229). Until now the detailed cross-talk between immune and skeletal systems over bone regeneration remains unclear, further investigation on different types of infiltrating immune cells, as well as their mutual-regulations during bone regeneration, would help to understand the ultimate role of S1P-S1PR1 signaling in osteoimmunology. It could be presumed that this signaling takes part in the maintenance of the balance between bone resorption and formation under physiological conditions. Especially under inflammatory conditions, a question arises about whether the activated S1P-S1PR1 signaling would trigger osteogenesis in osteoblast-precursors, and it could be proposed that this signaling plays a role in the pathogenesis of inflammation-related bone sclerosis lesions, such as bone spurs in arthritis or sequestrum in osteomyelitis. Another question lies in the mechanism and outcome of S1P-S1PR1 mediated osteogenesis: it has been proved that S1P-S1PR1 leads to induced Wnt-β-catenin signaling pathway to improve osteoblast differentiation (96); however, if β-catenin induction continues, it would result in interrupted Notch signaling and therefore should interfere the terminal differentiation toward osteocytes, as it has been identified that Wnt and Notch pathways are mutually exclusive during osteogenesis; and the up-regulated Notch signaling plays indispensable roles in osteocyte differentiation, while Wnt signaling is more dominant during osteoblast differentiation (230). Also, S1P-S1PR1 activation will leads to the activation of mTOR signaling (166, 202). Although mTOR has been found to play decisive roles in the transition from pre-osteoblasts to osteoblasts (231–233), however, it acts as an inhibitor in the autophagy—an indispensable process in extracellular calcium deposition during mineralization (234–239). It could be presumed that S1P-S1PR1-Akt-mTOR signaling pathway should play positive roles during early stage osteoblast differentiation, however, the later stage osteocyte differentiation as well as mineralization might be affected; also, the quality of such mineralization might be abnormal or even pathological, as compared with the physiological ones.

In summary, S1P, a key coupling factor for osteoclasts and osteoblasts, plays a complex role in bone remodeling by targeting both osteoclastogenesis and osteogenesis. The immunomodulatory feature of S1P-S1PR1 signaling further indicates that favors the inflammatory cell phenotypes in the adaptive immune system (T cell subsets), while induces macrophage polarization toward the anti-inflammatory phenotype. This dual role in immune system indicates that S1P-S1PR1 signaling might take part in the maintenance of continuous bone turnover under physiological conditions, while lead to the pathogenesis of bone deformities during inflammation. Further investigation of the S1P-S1PR1 signaling pathway should help to get a better understanding about osteoimmunology and therefore benefit the clinical approach for inflammatory bone disorders.

## Author Contributions

All authors listed have made a substantial, direct and intellectual contribution to the work, and approved it for publication. LX involved in the concept and design of the article, wrote the manuscript. YZ involved in the conception and design of the article, reviewed the manuscript. TF assisted with manuscript preparation. KB reviewed the manuscript. YX involved in the conception and design of the article, and reviewed the manuscript.

### Conflict of Interest Statement

The authors declare that the research was conducted in the absence of any commercial or financial relationships that could be construed as a potential conflict of interest.

## Supplementary Material

Figure S1 The RANKL-RANK axis mediated osteoclastogenic signals. RANK is activated when combining with its ligand RANKL. Activated RANK then triggers the down-stream osteoclastogenic signaling cascades. Activated TRAF6 induces the MAPK, IKK, and NF-κB signaling, which eventually result in activation of NFATc1 and osteoclastogenesis. RANKL: receptor activator of nuclear factor factor-kappa B ligand. RANK, receptor activator of nuclear factor-kappa B; TRAF6, tumor-necrosis factor (TNF) receptor-associated factor 6; IKK, inhibitor of nuclear factor kappa-B kinase; MAPK, mitogen-activated protein kinase; NF-κB, nuclear factor kappa B; AP-1, activator protein1; ERK, extracellular signal regulated kinase; JNK, c-Jun N-terminal kinase; NFATc1, nuclear factor of activated T-cells, cytoplasmic 1.
