# Supplementary figures and images for: S1P-S1PR1 Signaling: the “Sphinx” in Osteoimmunology
Source: Front Immunol. 2019 Jun 25;10:1409. doi: 10.3389/fimmu.2019.01409 (PMC6603153; doi:10.3389/fimmu.2019.01409)

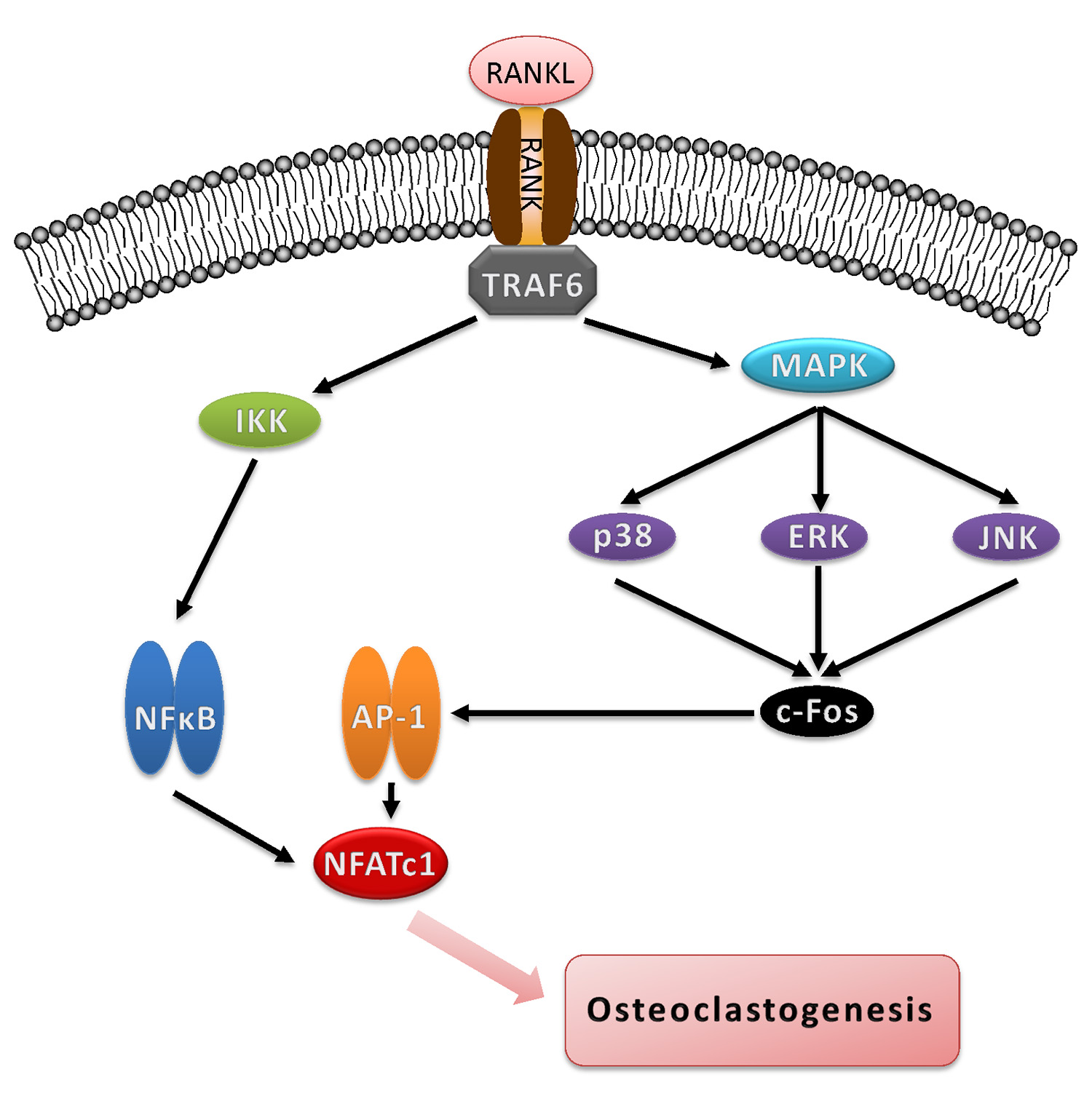

Supplement: Figure S1 — The RANKL-RANK axis mediated osteoclastogenic signals. RANK is activated when combining with its ligand RANKL. Activated RANK then triggers the down-stream osteoclastogenic signaling cascades. Activated TRAF6 induces the MAPK, IKK, and NF-κB signaling, which eventually result in activation of NFATc1 and osteoclastogenesis. RANKL: receptor activator of nuclear factor factor-kappa B ligand. RANK, receptor activator of nuclear factor-kappa B; TRAF6, tumor-necrosis factor (TNF) receptor-associated factor 6; IKK, inhibitor of nuclear factor kappa-B kinase; MAPK, mitogen-activated protein kinase; NF-κB, nuclear factor kappa B; AP-1, activator protein1; ERK, extracellular signal regulated kinase; JNK, c-Jun N-terminal kinase; NFATc1, nuclear factor of activated T-cells, cytoplasmic 1. [file Image_1.JPEG]
